# Factors That Impact the Psychological Wellbeing of Airborne Isolated Patients

**DOI:** 10.7759/cureus.12111

**Published:** 2020-12-16

**Authors:** Arwa Alghamdi, Amr Albanna, Sama Bukhari, Abeer Nafadi, Shaima Alharazi

**Affiliations:** 1 Medicine, King Saud bin Abdulaziz University for Health Sciences, Jeddah, SAU; 2 Medicine, King Saud bin Abdulaziz University for Health Sciences, King Abdullah International Medical Research Center, Jeddah, SAU

**Keywords:** airborne isolation, isolation, anxiety, depression

## Abstract

Background

Lower respiratory tract infections are one of the international leading causes of lost disability-adjusted years, and hence prevention measures, including isolation of high-suspect patients, were established to reduce the risk of transmission. However, isolation can negatively affect the psychological health of isolated patients, leading to anxiety or depression. The study aim was to investigate the association between types of isolation and the levels of anxiety and depression. Other factors that negatively influence the psychological status were identified.

Methods

This was an observational analytical cross-sectional study. The study included patients aged 18 years and above who had been isolated for at least 48 hours at King Abdulaziz Medical City in Jeddah, in the western region of Saudi Arabia. The data was gathered by interviewing the patients using the Hospital Anxiety and Depression Scale (HADS).

Results

Among the included 97 isolated patients, 52 (53%) were men, and 45 (47%) were women with a mean age of 49.39 ± 1.87. Among all participants, 70% were married, and 72% had children. The means of anxiety and depression scores were 5.08 ± 0.39 and 7.48 ± 0.40, respectively. There was no significant association between HADS and types of isolation (P=0.550). Female gender was significantly associated with abnormal HADS (36% vs. 17%; P=0.040). On the other hand, the frequency of physician follow-up visits reduced the risk for abnormal HADS (22% vs. 50%; P=0.040)

Conclusion

Just over one-fourth of the isolated patients had raised anxiety or depression scores. A trend to higher HADS was observed in airborne isolated patients. We found also that females were more susceptible to anxiety and depression, while frequent physician visits improved the psychological wellbeing of isolated patients.

## Introduction

Infectious diseases (ID) are defined as illnesses caused by contagious pathogenic microorganisms that can be transmitted from one infected patient to another healthy person [1]. Internationally, the second leading cause of lost disability-adjusted life years is lower respiratory tract infections, and diarrheal diseases and HIV/AIDS are the fourth and fifth, respectively; thus, protocols and prevention measures should be established to reduce their risks [2]. According to the guidelines for isolation precautions in hospitals published by the Centers for Disease Control and Prevention (CDC), patients who are at high suspicion for infectious diseases must start the standard precautions and isolation after the admission immediately until the culture reveals the underlying microorganism if present [3]. Contact isolation precautions are applied for organisms that transmit through direct or indirect contact with the patient or the patient’s surroundings. Meanwhile, droplet precautions are intended for pathogens that spread through respiratory secretions. However, for pathogens that remain suspended in the air for long times, airborne precautions are mandatory [4]. Although isolation is indispensable for infection control and to effectively restrain the transmission of the diseases, previous research has reported that it negatively affects the psychological health of isolated patients, leading to anxiety and depression. A systematic review described anxiety and depression as one of the adverse effects of contact isolation [5]. Furthermore, a study conducted in Nigeria showed that prolonged isolation has a significant impact on the psychosocial wellbeing of isolated patients, while a cross-sectional matched cohort study in the Netherlands demonstrated that short-term isolation does not affect anxiety and depression levels [6-7]. Several factors have been suggested as contributors to the depressive and anxious states of isolated patients, including separation from their marital partner, the apprehension that people know they have a serious contagious disease, and inability to participate in daily social and economic activities [6]. In conclusion, these studies emphasize that depression and anxiety are common among all types of isolation precautions. However, no study has compared the type of isolation to the extent of anxiety and depression levels yet. Therefore, this study aimed to investigate the association between types of isolation and the levels of depression and anxiety. We expected that airborne isolation has a higher influence on depression and anxiety scores compared to other types of isolation. Moreover, other factors that negatively influence the psychological status of isolated patients were identified.

## Materials and methods

Study design and setting

This was an observational analytical cross-sectional study among isolated patients at King Abdulaziz Medical City (KAMC) wards in Jeddah, in the western region of Saudi Arabia.

Sample size and sampling technique

The estimated sample size required to determine an increase in the prevalence of anxiety from 20% among patients admitted for non-infectious disorders to 40% among those admitted to rule out infectious diseases, with a power of 80% and alpha of 0.05 was around 182 subjects. The required sample size was estimated at the 95% confidence interval (CI) level with a 50% response distribution and a margin of error of ± 5%. This study followed a non-probability consecutive sampling technique.

Study subjects

Patients were eligible for the study inclusion criteria if they were aged 18 years and above and had been isolated for at least 48 hours to airborne, contact, or droplet isolation either for suspected or confirmed infection. However, we excluded patients who had previously been diagnosed with any underlying psychiatric disorder and patients on palliative care. Also, patients who could not understand Arabic or English or were unable to communicate with the interviewers, such as intubated and delirious patients, were excluded.

Data collection

**Figure 1:**
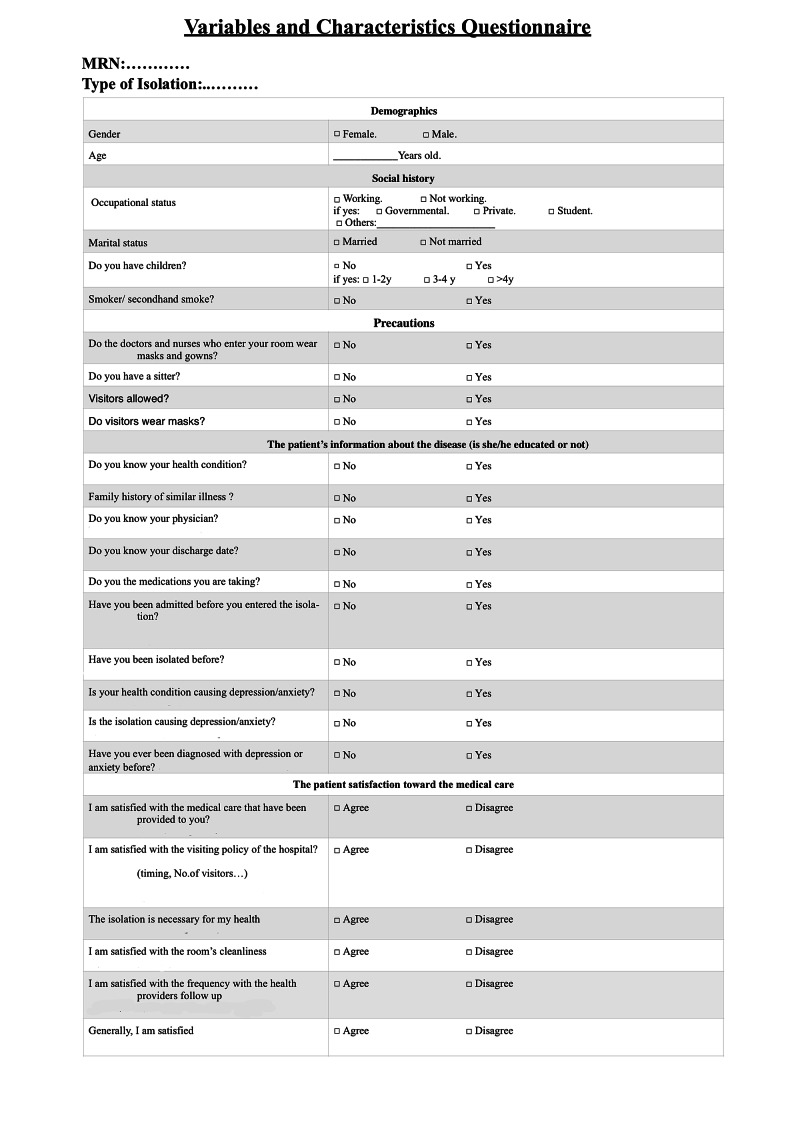
Variables and Characteristics Questionnaire

**Figure 2:**
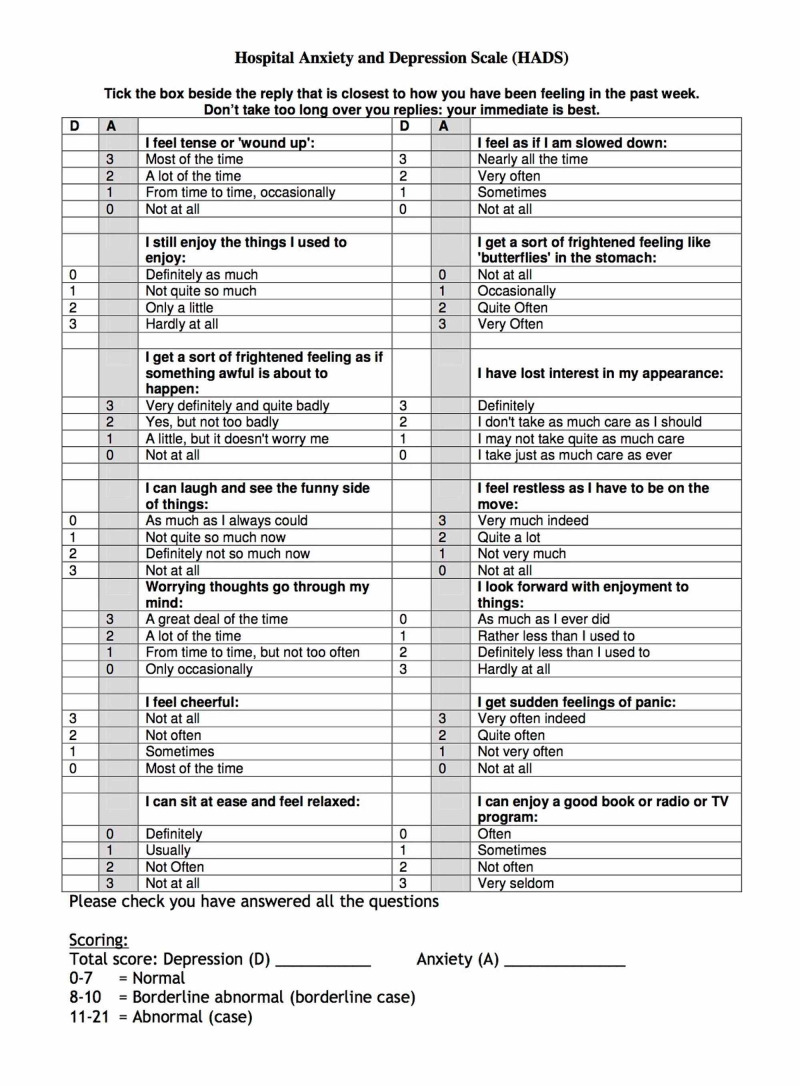
Hospital Anxiety and Depression Scale (HADS)

This study was approved by the International Review Board (IRB), King Abdullah International Medical Research Center (KAIMRC; approval SP17/273/3). All the investigators and data collectors have had the “RIGHT CARE, RIGHT NOW” Infection Prevention and Control Training & Competency Program, a course that prepares health care providers on applying suitable personal precaution equipment to access the isolation rooms with minimal risk of infection. We collected data by interviewing the participants after gaining their consent, and medical files were reviewed to confirm the diagnosis and length of isolation. We gathered data by using two datasheets: the variables and characteristics questionnaire, and the Hospital Anxiety and Depression Scale (HADS) (Figure 1, 2) [8].

The variables and characteristics questionnaire focused on patients’ demographics, patients’ information about the disease, and patients’ overall satisfaction toward the medical care provided. HADS is considered a validated and standardized psychological screening method to assess generalized anxiety in hospitalized patients. It consists of two groups of 14 questions; anxiety and depression separately. Each question was answered with a Likert scoring system: not at all (0 points), several days (1 point), more than half the days (2 points), nearly daily (3 points). The sum of the score is categorized into normal (0-7), borderline (8-10), and a score of (11-21) points out to significant psychological morbidity. In this study, the range (11-21) was considered the cutoff of abnormal results.

Data analysis and statistics

Microsoft Office Excel software was used for data entry and STATA 12 software (StataCorp., College Station, TX, USA) for data analysis. The proportion and mean for dichotomous and continuous variables, respectively, were measured to describe patients’ characteristics. χ2 test was used for comparative analysis. We considered a P-value of less than 0.05 to be significant.

## Results

Among the 130 subjects interviewed, 97 were included. Fifty-two (53%) were men and 45 (47%) were women with a mean age of 49.39 ± 1.87. Among all participants, 70% were married and 72% had children. The means of anxiety and depression scores of the study sample were 5.08 ± 0.39 and 7.48 ± 0.40, respectively (Table 1, 2).

**Table 1:** Characteristics of Participants (n= 97) SD: Standard Deveiation

Characteristics	Mean ± SD	95% Confidence Interval
Age (year)	49.39 ± 1.87	45.67% -53.11%
Anxiety score	5.08 ± 0.39	4.30% - 5.86%
Depression score	7.48 ± 0.40	6.69% - 8.28%

**Table 2:** Socio-demographics of the participants

Characteristics	Percentage	95% Confidence Interval
Females	46.88%	35.8% - 56.2%
Married	70.10%	59.57% - 78.75%
Having children	72.16%	61.78% - 80.58%

**Figure 3:**
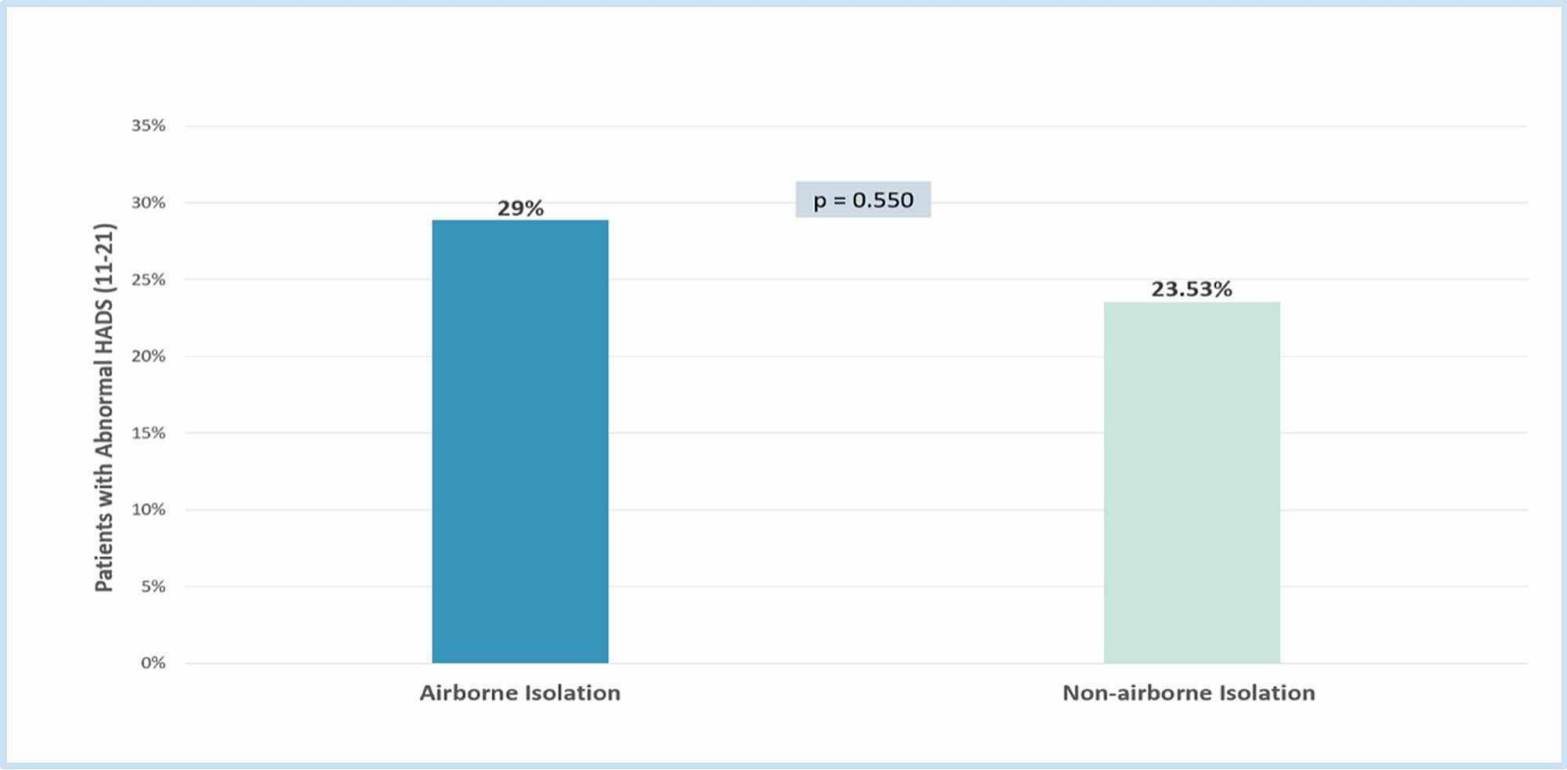
Anxiety and depression scores stratified by type of isolation HADS: Hospital Anxiety and Depression Scale

Figure 3 demonstrates that there was no significant difference between HADS among types of isolation (P = 0.550).

Among sociodemographic factors, gender was the only factor that showed significant association with HADS, as 36% of females and 17% of males had abnormal anxiety or depression scores (P = 0.040) (Table 3).

**Table 3:** Association between participants characteristics and abnormal HADS HADS: Hospital Anxiety and Depression Scale

Factor	Total	Abnormal HADS	P-value
Gender			
Females	45	16 (35.56%)	0.04
Males	52	9 (17.31%)	
Occupational status			
Employed	28	5 (17.86%)	0.256
Unemployed	69	20 (28.99%)	
Having children			
Yes	70	16 (22.86%)	0.29
No	27	9 (33.33%)	
Smoking			
Yes	34	11 (32.35%)	0.297
No	62	14 (22.58%)	
History of previous admission			
Yes	44	7 (15.91%)	0.058
No	52	17 (32.69%)	
History of previous isolation			
Yes	37	11 (29.73%)	0.484
No	60	14 (23.33%)	

**Figure 4:**
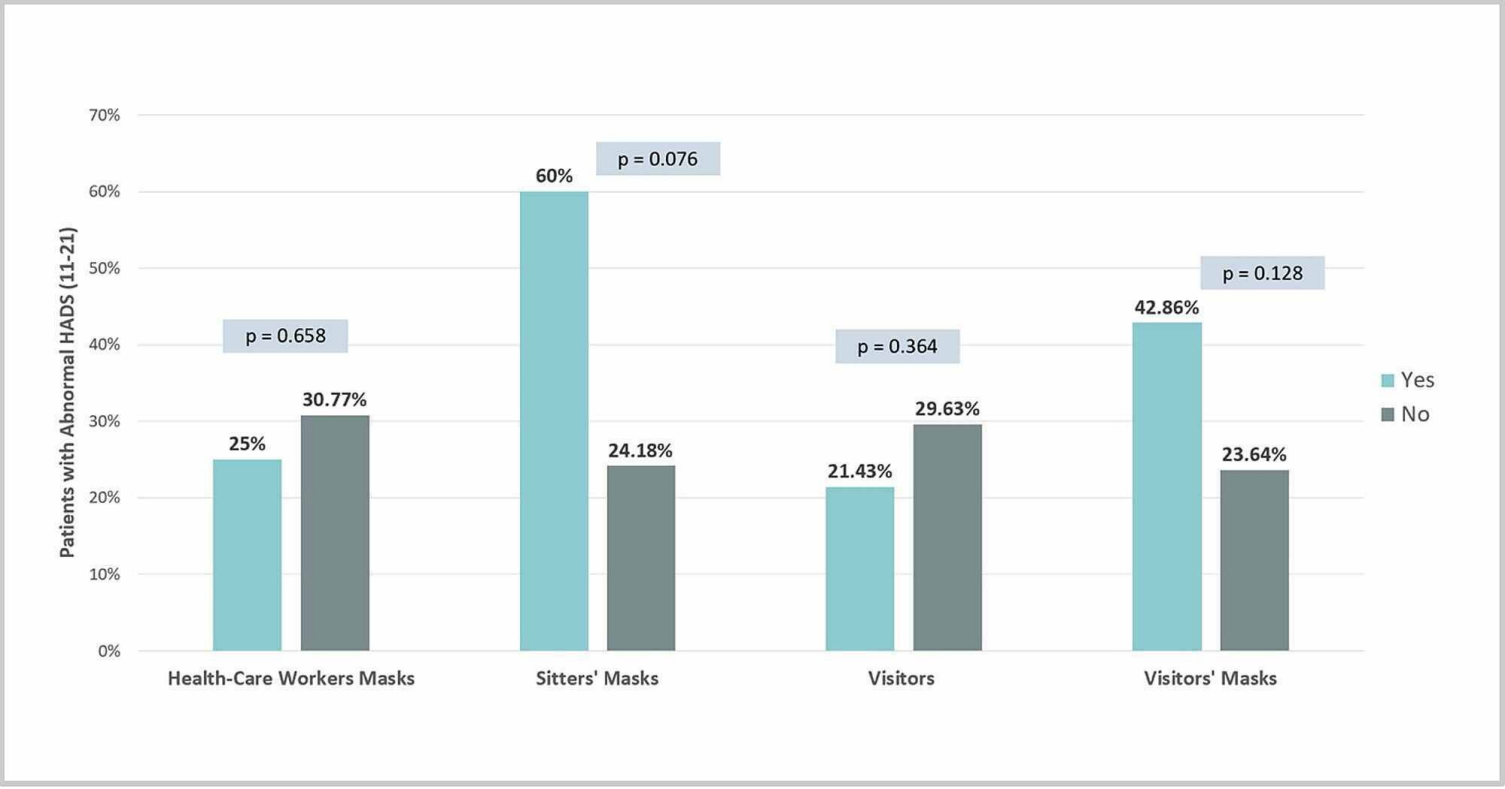
Isolation precautions related factors HADS: Hospital Anxiety and Depression Scale

None of the isolation precaution-related factors were significantly associated with HADS; however, there was a trend toward lower HADS among patients who had visitors (Figure 4).

**Figure 5:**
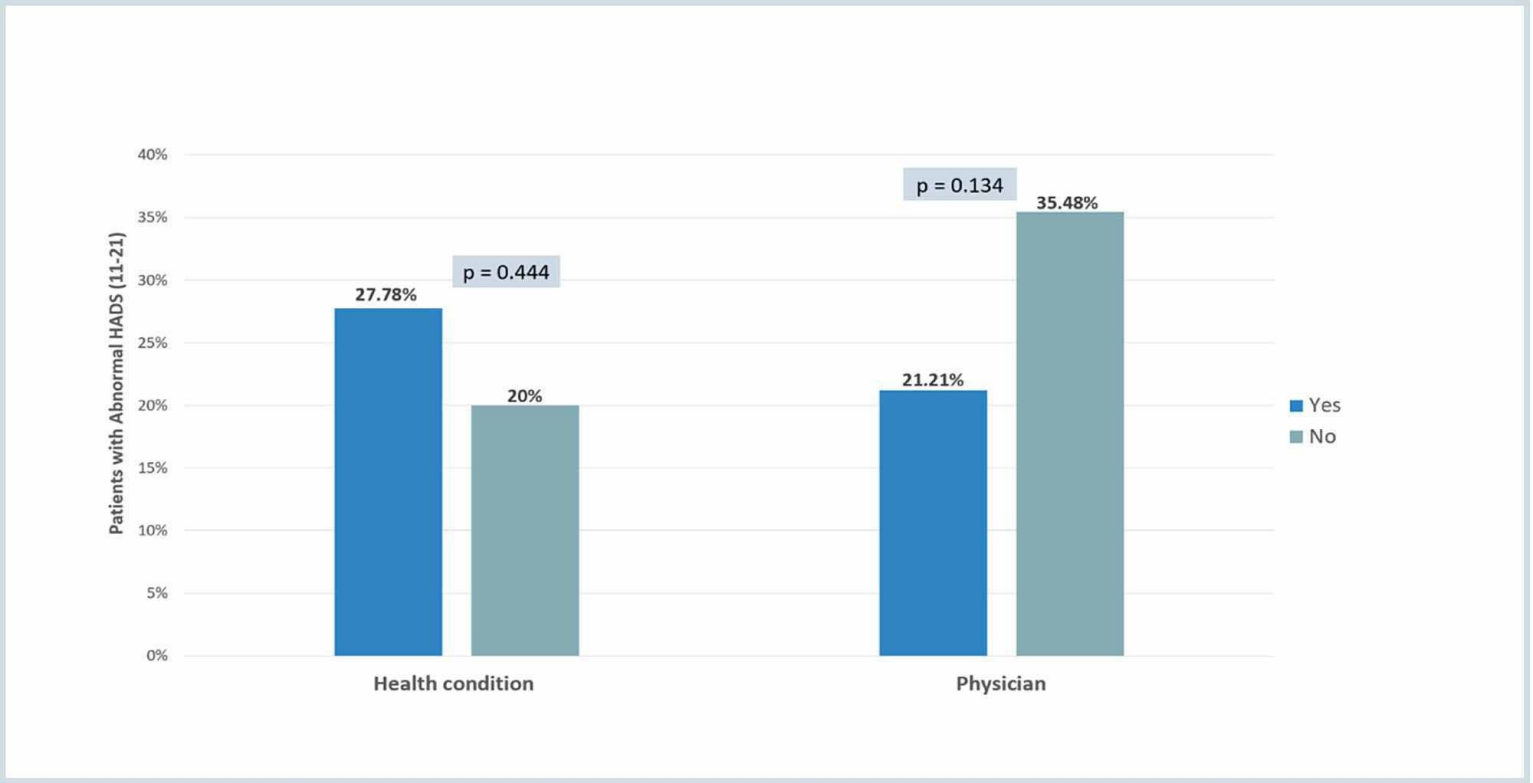
Patient education-related factors HADS: Hospital Anxiety and Depression Scale

Figure 5 shows that there was no statistically significant association between HADS and patient education-related factors, including patients' knowledge about the health condition and physician.

**Figure 6:**
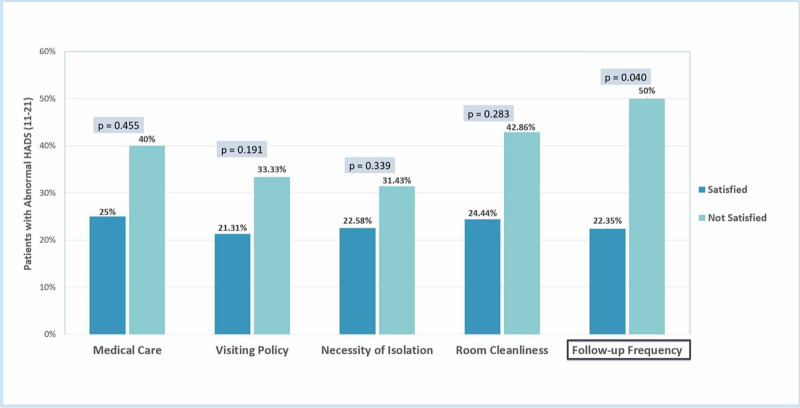
Patients satisfaction related factors HADS: Hospital Anxiety and Depression Scale

Satisfaction towards the frequency of physician follow-up visits significantly reduced the risk of abnormal HADS (22% vs. 50%; P = 0.040) (Figure 6).

## Discussion

This cross-sectional study aimed to investigate the effect of airborne isolation on the levels of anxiety and depression of isolated patients. It is well established that isolation is an effective measurement to control infectious diseases, however, research suggests that it negatively affects the psychological wellbeing of the patients [9-10]. This was demonstrated through our findings as more than one-fourth (26%) of the participants had raised anxiety or depression scores. A study that was conducted on isolated patients reported significant anxiety in 46% of the patients using the HADS [9]. The difference between the percentages is attributable to the fact that they considered borderline and abnormal ranges to be significant, while only abnormal ranges were considered in our study. Airborne isolation was expected to have higher anxiety and depression levels. The findings showed a trend to higher anxiety or depression among airborne isolated patients, and that is mainly due to the strict precaution strategies and visiting policy.

Our study demonstrated a substantial relationship between female gender and abnormal anxiety or depression scores. This was supported by a study conducted in Edirne, Turkey in 2012 among contact isolated patients, which commented that isolated female patients experienced more psychological morbidity and are more prone to develop depression compared to male patients [10]. Depression and anxiety are experienced differently among opposite genders with various perceptions. According to a psychological study in this interest, women are two times more susceptible to depression than male patients [11]. This higher risk of anxiety and depression among isolated females can be explained due to the separation from their children, which attributes strongly to their emotional state. Correspondingly, we found that subjects who have children had a higher tendency toward abnormal HADS. This may be due to the parents’ concerns about directly providing their children’s care. This is supported by a Nigerian study, which indicates that subjects were more susceptible to poor psychological wellbeing when their children’s needs were provided by others compared to themselves [6].

We also found a trend toward higher anxiety or depression scores in isolated patients who were unemployed. However, this opposes what was observed in the Nigerian study where employed respondents were eight times more likely to experience poor psychosocial wellbeing compared to unemployed respondents [6]. This could be explained by considering the poverty and economic instability that the Nigerian population encounters. Therefore, a greater psychological burden is experienced by these patients when health issues threaten their careers and, thus, socioeconomic status. On the other hand, living in a stable environment economically for employed individuals but with a high workload could contribute to the result mentioned in our study. 

Among isolation precaution-related factors, we found a trend toward lower HADS scores when visitors were allowed to enter the patients' rooms. Similarly, a study conducted in the Philippines reports a significant association between low perceived social support and depression among tuberculosis patients [12]. These patients who tend to express higher rates of depression or anxiety, especially when isolated, need the support of their family members and friends to improve psychological wellbeing. Such family support, however, cannot be always guaranteed for airborne isolated patients; therefore, health care providers should consider additional measures to support their psychosocial wellbeing.

Another significant association was observed between patient satisfaction and anxiety and depression levels. Isolated patients who were not satisfied with their physician follow-up frequency had a significant increase in anxiety or depression levels. This was demonstrated as well in a study that assessed the satisfaction among isolated patients compared to non-isolated hospitalized patients, in which they found significant dissatisfaction with health care workers' availability and visits [9]. We hypothesize that patients who do not have the opportunity to convey their concerns to their physicians or do not have answers to their questions regarding their health conditions may also experience more anxiety or depression.

The present study has some limitations. First, the studied population may not adequately represent the general hospital population. This could be due to the limited number of wards and isolation rooms in the studied center, and the exclusion of subjects with language and communication barriers. As a result, this restricted the power of this study to detect more influencing factors.

## Conclusions

Over one-fourth of the isolated patients had abnormal anxiety or depression scores. A trend to higher HADS was observed in airborne isolated patients. We found also that females were more susceptible to anxiety and depression, while frequent physician follow-up visits improved the psychological wellbeing of isolated patients. 

We recommend providing a proper psychological assessment for high-risk patients who require isolation. Future longitudinal studies in multiple centers and over longer periods are recommended to evaluate a variety of subjects and assess the influencing factors for each type of isolation individually.
